# Case Report: ECMO rescue for cardiac arrest in a hyperthyroid patient triggered by COVID-19 infection

**DOI:** 10.3389/fcvm.2025.1642892

**Published:** 2025-10-14

**Authors:** Shuaiting Ma, Lutao Xie, Pin Lan, Haohao Wu, Mingjun Liu, Yi Chen

**Affiliations:** Department of Emergency Medicine, The Fifth Affiliated Hospital of Wenzhou Medical University, Lishui Central Hospital, Lishui Hospital of Zhejiang University, Lishui, Zhejiang, China

**Keywords:** fulminant myocarditis, thyroid storm, SARS-CoV-2, VA-ECMO, CRRT

## Abstract

**Background:**

Coronavirus disease 2019 (COVID-19) is a global pandemic, while both fulminant myocarditis (FM) and thyroid storm (TS) are life-threatening critical conditions. When these three conditions coexist in a single patient, the survival outcome can be severely compromised.

**Case introduction:**

We report a case of cardiac arrest in a hyperthyroid patient triggered by COVID-19 infection. The patient developed symptoms such as fatigue, chest tightness, and chest pain following SARS-CoV-2 infection, and experienced respiratory and CA en route to the hospital. Ultimately, the patient was diagnosed with FM and TS. The patient was treated with veno-arterial extracorporeal membrane oxygenation (VA-ECMO) combined with continuous renal replacement therapy (CRRT). After hemodynamic stabilization, the patient received pharmacological management for arrhythmia control and hyperthyroidism, along with other symptomatic treatments. The patient eventually recovered and was discharged.

**Conclusion:**

In hyperthyroid patients infected with SARS-CoV-2, clinicians should remain vigilant for the potential development of FM and TS. VA-ECMO combined with CRRT represents an effective therapeutic approach in such critical scenarios.

## Introduction

Coronavirus disease 2019 (COVID-19) infection can lead to multi-organ dysfunction; however, fulminant myocarditis (FM) induced by SARS-CoV-2 is relatively rare (1). It progresses rapidly and is life-threatening, with a mortality rate exceeding 50% (2, 3). Thyroid storm (TS), a severe complication of hyperthyroidism, is often triggered by infections, surgery, or trauma, with a mortality rate as high as 10%–30% (4, 5).

We describe a rare case of a patient who developed both FM and TS following COVID-19 infection. The patient had a history of hyperthyroidism and experienced persistent fatigue for two weeks after SARS-CoV-2 infection. During hospitalization, the patient suffered respiratory and cardiac arrest (CA). Through comprehensive treatment, including veno-arterial extracorporeal membrane oxygenation (VA-ECMO) combined with continuous renal replacement therapy (CRRT), the patient eventually recovered and was discharged.

## Case report

A 47-year-old female with a history of hyperthyroidism, on regular medication but without routine thyroid hormone monitoring, denied any history of hypertension, diabetes, or heart disease. She presented with “Fatigue for 2 weeks since COVID-19 diagnosis” at a local hospital, two weeks prior, she had contracted COVID-19, after which she experienced persistent fatigue and recent diarrhea. Initial evaluation at the local hospital showed no significant abnormalities in cardiac enzymes or electrocardiogram (ECG). During the hospitalization, the patient suddenly developed respiratory and CA. Immediate endotracheal intubation and continuous cardiopulmonary resuscitation (CPR) were initiated. CPR was unsuccessful, and the patient did not return of spontaneous circulation after 30 min of advanced life support. The physicians at the local hospital held a conference with the family, who expressed their desire for continued aggressive resuscitation efforts. Consequently, the local medical team contacted our institution to request assistance from our extracorporeal membrane oxygenation (ECMO) team. The ECMO circuit was successfully established 3 h after the onset of CA. The patient was subsequently transferred to the emergency intensive care unit (EICU) at our institution 7 h post-arrest.

**Figure 1:**
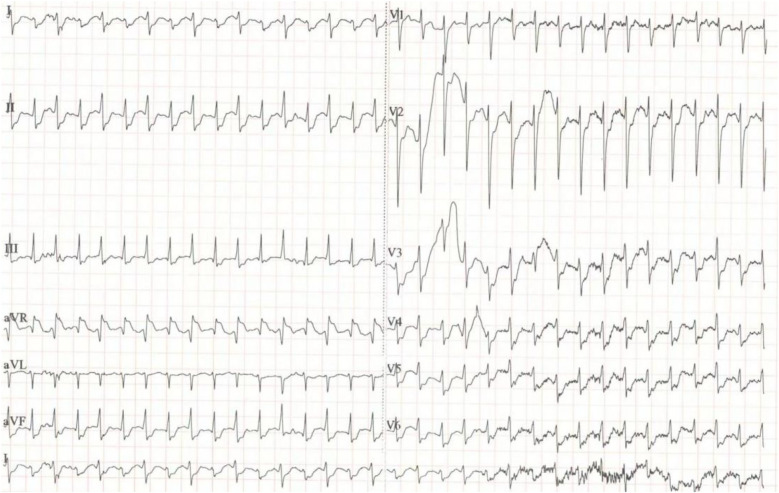
The patient's ECG.

At the time of admission: Blood tests: WBC 25.2 × 10^9^/L, hemoglobin 96 g/L, PLT 174 × 10^9^/L. Arterial blood gas: pH 7.25, PaCO_2_ 33.0 mmHg, PaO_2_ 150.0 mmHg, lactate >15 mmol/L. Biochemistry: ALT 1,913 U/L, AST 2,892 U/L, creatinine 102 μmol/L. Cardiac markers: Troponin >80 ng/ml, myoglobin >2,000 ng/ml. SARS-CoV-2 nucleic acid test: Positive. ECG: Supraventricular tachycardia, heart rate 200 bpm, no significant ST-segment changes (Figure 1). Echocardiography: Diffuse hypokinesia, left ventricular ejection fraction (LVEF) 35%.CT angiography of the lungs, thoracic aorta, and coronary arteries, as well as brain CT: No significant abnormalities. After 7 h of adequate fluid resuscitation, the patient's urine output remained less than 100 ml. Based on the patient's recent viral infection, elevated troponin levels, and diffuse cardiac hypokinesia, FM was highly suspected according to the 2024 ACC Expert Consensus Decision Pathway on Strategies and Criteria for the Diagnosis and Management of Myocarditis (6). Other diagnoses included CA, acute kidney injury (AKI), metabolic acidosis (pH 7.25), and hyperthyroidism.

**Figure 2:**
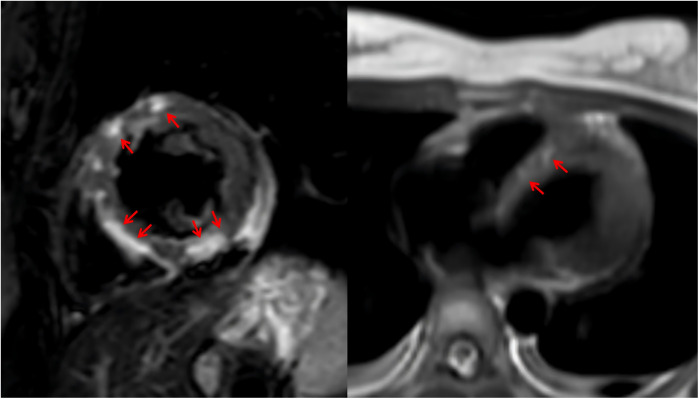
The patient's MRI.

**Figure 3:**
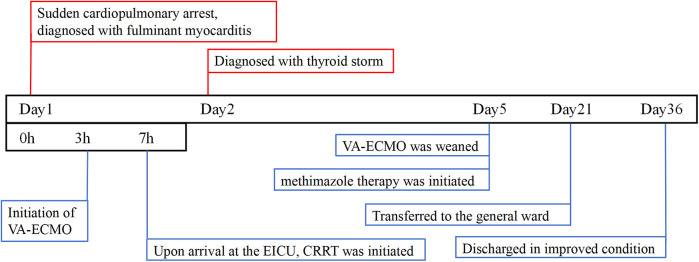
Patient's treatment course.

Given the onset of AKI, CRRT was initiated immediately upon the patient's arrival in the emergency intensive care unit (EICU) to manage fluid overload, correct severe metabolic acidosis, and remove inflammatory mediators. The CRRT modality selected was continuous venovenous hemofiltration (CVVH). Upon admission, continuous cardiac monitoring revealed frequent episodes of supraventricular tachycardia. After hemodynamic stabilization, a low-dose esmolol infusion was administered to control the heart rate. Initial thyroid function tests showed the following results: triiodothyronine (T3) 5.49 ng/ml, free triiodothyronine (FT3) >20.00 pg/ml, thyroxine (T4) 15.78 μg/dl, free thyroxine (FT4) >5.00 ng/dl, and thyroid-stimulating hormone (TSH) 0.070 μIU/ml. The patient exhibited elevated FT3 and FT4 levels, based on both the Japan Thyroid Association (JTA) guidelines for the management of thyroid storm and the Burch-Wartofsky Point Scale (65 points), these findings are consistent with a diagnosis of TS (7). A review of the relevant literature indicated that CRRT could be utilized in the acute phase management of TS (8). The CRRT modality was not adjusted. Consequently, the decision was made to continue CRRT, with its efficacy being assessed through serial monitoring of thyroid hormone levels. By day 3, thyroid hormone levels significantly decreased (Table 1). By day 5, LVEF improved to 57%, and no ventricular arrhythmias were observed. Unfortunately, both the ultrasonography and ECG were performed at the bedside using mobile devices. Due to a routine memory update of the equipment, the corresponding imaging data were not saved. VA-ECMO was successfully weaned. By day 6, methimazole was added to manage hyperthyroidism. After 3 weeks of treatment, the patient was successfully transferred to the general ward and underwent cardiac MRI, which revealed multifocal myocardial edema involving the left ventricular interventricular septum, anterior wall, and inferior wall (Figure 2). After 36 days of systematic treatment, the patient was successfully discharged with complete renal function recovery (no longer requiring renal replacement therapy) and satisfactory neurological recovery, demonstrating full independence in daily activities and achieving an optimal clinical outcome. Figure 3 is a timeline of the clinical condition progress and major management of the patient.

**Table 1:** Thyroid hormone levels over time in a patient.

Date	Day 2	Day 3	Day 8	Day 12	Day 14
T3 (ng/ml)	5.49	1.78	0.93	0.39	0.35
T4 (μg/dl)	15.78	11.72	8.46	2.27	2.16
FT3 (pg/ml)	>20.00	7.93	3.95	1.87	2.15
FT4 (ng/dl)	>5.00	3.06	2.64	0.71	0.57
TSH (μIU/ml)	0.070	<0.004	0.010	<0.004	<0.004

## Discussion

COVID-19 primarily manifests with respiratory symptoms; however, recent studies have demonstrated its potential to affect multiple organ systems. Several reports have documented myocarditis in patients with SARS-CoV-2 infection (9). Additionally, a limited number of studies have described cases of TS associated with COVID-19 (10). The simultaneous occurrence of FM, TS, and CA triggered by SARS-CoV-2 infection is exceedingly rare, with no similar cases reported in the literature to date. FM is a severe cardiovascular condition characterized by acute onset, rapid progression, and a high risk of cardiogenic shock, malignant arrhythmias, or sudden death (11).

TS typically occurs in hyperthyroid patients under the influence of precipitating factors such as infection, surgery, or trauma, leading to severe metabolic and systemic disturbances (6). During TS, excessive thyroid hormone secretion exerts direct cardiotoxic effects, disrupting myocardial electrophysiology (12). If TS is not promptly controlled, the persistent hypermetabolic state and adverse effects of thyroid hormones on the myocardium can exacerbate myocardial injury, promote the development and progression of myocarditis, and further impair cardiac function, potentially resulting in acute heart failure or life-threatening arrhythmias (12). According to the 2016 JTA Guidelines for the Management of TS (7), this patient met the diagnostic criteria for TS. Based on the available evidence and diagnostic findings, it is challenging to definitively establish the causal relationship between SARS-CoV-2 infection, TS, and FM. However, it is clear that hyperthyroid patients infected with SARS-CoV-2 should be closely monitored for the development of FM and TS.

For patients with FM complicated by cardiogenic shock or life-threatening arrhythmias, mechanical circulatory support is crucial as it reduces cardiac workload and supports recovery. In this case, VA-ECMO was successfully initiated three hours after the patient experienced respiratory and CA. The delay was primarily due to the geographical challenges of our region, characterized by mountainous and hilly terrain, resulting in a two-hour transit time between hospitals. Additionally, the local hospital lacked ECMO capabilities and facilities. Despite the ECMO team's immediate response, the entire process took approximately three hours. This highlights the importance of equipping primary hospitals with ECMO capabilities and establishing a robust support network for CA in mountainous regions (13). Currently, VA-ECMO is widely used in the management of FM with circulatory failure. The favorable outcome in this patient, who experienced a three-hour CA, underscores the critical role of VA-ECMO support and suggests that prolonged resuscitation and ECMO support may be warranted for in-hospital CA patients.

According to clinical guidelines, the management of TS includes treating the underlying cause, inhibiting thyroid hormone synthesis and release, and controlling heart rate (4). For this patient, rapidly mitigating the adverse effects of thyroid hormones on the heart was a key therapeutic priority. Initially, CRRT was selected to address severe acidosis secondary to prolonged CPR and concurrent kidney dysfunction. Upon confirming TS, literature review revealed evidence supporting CRRT's efficacy in reducing serum T3 and T4 levels in TS patients (8). Consequently, CRRT was continued. By day 3 of hospitalization, the patient's T3 and T4 levels had significantly decreased, demonstrating the effectiveness of CRRT in lowering thyroid hormone levels. However, the patient initially experienced recurrent supraventricular tachycardia. Following hemodynamic stabilization, esmolol and propranolol were sequentially administered to control heart rate. By day 5, no further ventricular arrhythmias were observed. Liver function improved, and methimazole was added on day 6 to manage hyperthyroidism, with subsequent dose adjustments to pre-illness levels, resulting in well-controlled disease progression.

## Conclusion

Hyperthyroid patients with COVID-19 should be closely monitored for FM and TS. Early assessment of cardiac injury markers and function is essential to prevent severe complications like CA. VA-ECMO combined with CRRT represents an effective treatment strategy for patients with FM complicated by TS.

## Data Availability

The original contributions presented in the study are included in the article/Supplementary Material, further inquiries can be directed to the corresponding author/s.
